# Corneal biomechanical changes in allergic conjunctivitis

**DOI:** 10.1186/s40662-021-00241-7

**Published:** 2021-05-03

**Authors:** Qian Wang, Yuqing Deng, Saiqun Li, Xinyue Du, Xuan Zhao, Tingting Zhang, Jin Yuan

**Affiliations:** State Key Laboratory of Ophthalmology, Zhongshan Ophthalmic Center, Sun Yat-Sen University, Guangzhou, Guangdong China

**Keywords:** Corneal biomechanics, Allergic conjunctivitis, Corneal tomography, Corneal epithelial thickness mapping, Corneal densitometry

## Abstract

**Background:**

To explore corneal biomechanical changes, identify related factors and determine early indicators of keratoconus (KC) development risk in allergic conjunctivitis (AC) patients.

**Methods:**

A total of 50 patients, including 20 eyes without AC and 30 eyes with AC were enrolled in this study. All patients underwent a complete ocular examination, including evaluations of clinical manifestations of AC, corneal tomography and densitometry by Pentacam, corneal biomechanics by Corvis ST, and corneal and epithelial thickness mapping by RTvue optical coherence tomography (OCT).

**Results:**

The index of surface variance (ISV), index of vertical asymmetry (IVA), keratoconus index (KI), index of height decentration (IHD) and Belin/Ambrosio enhanced ectasia total deviation index (BAD-D) were significantly higher in the AC group than in the non-allergic conjunctivitis (NAC) group (*P* < 0.05). The tomography and biomechanical index (TBI) was also significantly higher in the AC group (*P* = 0.04). The average epithelial thickness in the 2–7 mm annulus was significantly thinner in the AC group than in the NAC group (*P* < 0.05). The average densitometry of the total cornea and the anterior layer were higher in the AC group than in the NAC group (*P* < 0.001). The ISV, IVA, KI, IHD and BAD-D were significantly correlated with the TBI and changes in corneal epithelial thickness in AC patients (*P* < 0.05). The changes in epithelial thickness were closely related to the eye rubbing frequency and allergic sign scores (*P* < 0.05).

**Conclusions:**

AC patients should be advised to routinely undergo corneal tomographic and biomechanical measurements, and the TBI could be used as an indicator of KC development risk in AC patients.

**Trial registration:**

Corneal Biomechanical Changes of Allergic Conjunctivitis, NCT04299399. Registered March 3, 2020 - Retrospectively registered.

## Background

Allergic conjunctivitis (AC), affecting approximately 15–40% of the global population, is one of the most common ocular surface diseases [1, 2]. There is emerging evidence for the close association between AC and keratoconus (KC). Several studies have found that KC patients have a higher prevalence of AC and a higher frequency of eye rubbing than non-KC subjects [3–6]. Furthermore, recent studies have identified eye rubbing, itchy eyes, and AC as risk factors for KC [6, 7]. In addition, AC has also been proven to exacerbate KC conditions [5, 8]. Mazzotta et al. [8] found that KC progression occurred more rapidly and to a greater extent in patients with concomitant allergy, eye rubbing and elevated matrix metalloproteinase 9 (MMP9) levels in tears and that KC progression was closely related to the severity of allergy.

KC is a degenerative disorder of the cornea that was first described as a noninflammatory ectatic disease, but recent studies have implicated that the action of inflammatory mediators are involved in KC development and progression [8–10]. Corneal injury and elevated inflammatory mediator release caused by excessive eye rubbing in AC patients have been proposed as possible mechanisms of KC development. The corneal microtrauma and thinning caused by mechanical rubbing leads to reduced corneal rigidity and corneal remodeling, resulting in increased corneal curvature [11, 12]. Moreover, it has been proven that AC and eye rubbing can elevate the concentrations of inflammatory molecules in tears, including MMPs, interleukin (IL)-4, IL-5, IL-10, interferon (IFN)-γ and tumor necrosis factor-α (TNF)-α, which are involved in keratocyte apoptosis and tissue remodeling [8, 11, 13, 14].

The typical characteristics of KC include progressive thinning of the corneal stroma and corneal protrusion. However, it has been proposed that corneal biomechanical alterations occur earlier than topographic changes in KC patients; thus, corneal biomechanical alterations can be used for the early diagnosis of KC [15–18]. Although few previous studies have shown that eye rubbing and vernal keratoconjunctivitis (VKC) can cause a reduction in corneal biomechanics [19, 20], the influencing factors and possible underlying mechanisms remain unclear. The aim of this study was to further confirm AC as an etiology of KC and to elucidate the early indicators of KC development risk in AC patients. We explored corneal biomechanical changes and identified the sensitive biomechanical indicators and the related factors in AC patients.

## Patients and methods

### Participants

This was a case-control study involving 20 non-allergic conjunctivitis (NAC) patients and 30 patients with AC who visited the Zhongshan Ophthalmic Center, Sun Yat-Sen University. The study was approved by the Ethics Committee of the study hospital (2020KYPJ008) and adhered to the tenets of the Declaration of Helsinki. This trial is registered at ClinicalTrials.gov as NCT04299399. All patients (or the patient’s legal guardian) provided informed consent before the study.

The diagnosis of AC was based on clinical symptoms (itching, redness, foreign body sensation or increased mucous discharge) and signs (conjunctival hyperemia, swelling, palpebral conjunctival papillae, limbal swelling or Horner-Trantas dots) specific for AC. Only patients with an allergic history of more than 2 years were included. NAC patients were free from any other ophthalmic or systemic disease, except for refractive error. To eliminate the potential effect of high refractive error on corneal biomechanics, patients with high refractive errors (spherical diopter > 6 D and/or cylinder diopter > 2 D) were excluded. Other exclusion criteria include: 1) other active ocular inflammatory diseases or corneal scarring; 2) history of ocular surgery or trauma; 3) systemic diseases such as immune diseases or connective tissue diseases; 4) use of a soft contact lens within 2 weeks or a rigid contact lens within 1 month; and 5) inability to fixate or cooperate. The examinations were performed during the chronic inflammation stage in AC patients, and each measurement was obtained by a single examiner.

### Evaluation of clinical manifestations in AC patients

The eye rubbing frequency and ocular allergic sign scores were evaluated in AC patients. The eye rubbing frequency was assessed on a scale ranging from 1 to 5, where 1 represents no eye rubbing, and 5 represents constant eye rubbing [3, 21]. Signs of conjunctival hyperemia, swelling, papillae, and corneal epithelial disorder were assessed and graded by severity from 0 (no corresponding signs) to 3 (serious signs) by the clinician [22–24].

### Corneal topography and densitometry

The corneal morphological parameters measured by Pentacam (Oculus, Inc., Wetzlar, Germany) included central corneal thickness (CCT) at the corneal apex, corneal keratometric indices [the keratometry of the flattest and steepest meridian in the center (K1 and K2), mean central keratometry (Km) and maximum keratometry (Kmax)], topometric indices [index of surface variance (ISV), index of vertical asymmetry (IVA), keratoconus index (KI), central keratoconus index (CKI), index of height asymmetry (IHA) and index of height decentration (IHD)], and the Belin/Ambrosio enhanced ectasia total deviation index (BAD-D). In addition, corneal densitometry of four different annuli (0–2 mm, 2–6 mm, 6–10 mm and 10–12 mm) and three different corneal layers (anterior, central, and posterior) was performed using the densitometry function of the Pentacam Scheimpflug system.

### Corneal biomechanics

The main corneal biomechanical parameters provided by Corvis ST (Oculus, Inc., Wetzlar, Germany) include intraocular pressure (IOP), biomechanical-corrected IOP (bIOP), CCT, first applanation (A1) time, A1 velocity, A1 length, second applanation (A2) time, A2 velocity, A2 length, deformation amplitude (DA), highest concavity (HC) time, peak distance (PD), HC radius, max DA ratio (2 mm), max DA ratio (1 mm), Ambrosio relational thickness horizontal (ARTh), stiffness parameter at first applanation (SP-A1), Corvis biomechanical index (CBI) and tomography and biomechanical index (TBI).

### Corneal and epithelial thickness mapping

Corneal and epithelial thickness mapping was performed by RTvue optical coherence tomography (OCT; Optovue Inc., Fremont, CA) with a corneal adapter module lens. The “PachymetryWide” scan pattern was chosen to scan an area 9 mm in diameter. The corneal and epithelial thicknesses of four concentric radial areas (0–2 mm, 2–5 mm, 5–7 mm and 7–9 mm) were displayed on the map. Other pachymetry parameters included the minimum (min) and the difference between the minimum and maximum (min-max) within a diameter of 5 mm. Other epithelial parameters included the min, max, min-max and standard deviation (SD) of the epithelial thickness within a diameter of 7 mm.

### Statistical analysis

Only one eye of each patient was selected for analysis to avoid bias (the eye with the more severe condition was selected in AC patients, while one eye was randomly selected in NAC patients). Statistical analyses were performed using SPSS version 25.0 (SPSS, Inc., Chicago, Illinois, USA). All continuous variables were tested for normality by the Kolmogorov–Smirnov test. Data with a normal distribution are expressed as the mean ± SD, unless reported as the median (interquartile range). The independent samples t-test and Mann-Whitney U test were used to evaluate differences between two groups of normally distributed variables or non-normally distributed variables, respectively. Categorical variables were compared using the χ^2^ test. Spearman’s correlation test was used to determine correlations between data with a skewed distribution, ordinal categorical variables or ranked ordinal data. A *P* value of less than 0.05 is considered to be statistically significant.

## Results

### Patient characteristics

Twenty eyes in 20 NAC patients and 30 eyes in 30 AC patients were included in this study. Table 1 shows the demographics of the included patients. There was no significant difference in the mean age, sex ratio or IOP (measured by Corvis ST) between the NAC and AC groups (*P* > 0.05 for all).

**Table 1:** Demographics of the non-allergic conjunctivitis (NAC) and allergic conjunctivitis (AC) groups

Characteristic	NAC	AC	*p*
	(*n* = 20)	(*n* = 30)	
Age, mean ± SD (years)	18.75 ± 7.99	19.37 ± 10.59	0.83^*a*^
Sex, male, n (%)	13 (65.0%)	20 (66.7%)	0.90^*b*^
IOP (mmHg)	16.03 ± 2.40	15.90 ± 1.96	0.84^*a*^

*NAC* = non-allergic conjunctivitis; *AC* = allergic conjunctivitis; *IOP* = intraocular pressure; *SD* = standard deviation

^*a*^ Independent samples t-test

^*b*^ χ^2^ test

### Corneal tomography

The main corneal tomographic parameters of the NAC and AC groups are shown in Table 2. The ISV, IVA, KI, IHD and BAD-D in the AC group were significantly higher than those in the NAC group (*P* < 0.05 for all). There was no significant difference observed in the CCT, K1, K2, Km, Kmax, CKI or IHA between the two groups (*P* > 0.05 for all).

**Table 2:** Comparison of corneal tomographic parameters between the non-allergic conjunctivitis (NAC) and allergic conjunctivitis (AC) groups

Parameter	NAC	AC	*p*^*a*^
	Mean ± SD	Mean ± SD	
CCT (μm)	550.45 ± 21.79	555.80 ± 25.89	0.45
K1 (D)	42.43 ± 1.05	42.50 ± 1.27	0.85
K2 (D)	43.40 ± 1.08	43.91 ± 1.45	0.18
Km (D)	42.90 ± 1.03	43.20 ± 1.28	0.38
Kmax (D)	43.90 ± 1.14	44.52 ± 1.51	0.13
ISV	16.50 ± 4.21	23.40 ± 10.35	0.01
IVA	0.14 ± 0.04	0.19 ± 0.10	0.04
KI	1.03 ± 0.02	1.05 ± 0.03	0.02
CKI	1.01 ± 0.00	1.01 ± 0.01	0.07
IHA	5.05 ± 4.16	6.75 ± 4.04	0.16
IHD	0.01 ± 0.00	0.02 ± 0.01	0.03
BAD-D	0.88 ± 0.37	1.41 ± 0.59	0.001

*NAC* = non-allergic conjunctivitis; *AC* = allergic conjunctivitis; *CCT* = central corneal thickness; *K1* = keratometry of the flattest meridian; *K2* = keratometry of the steepest meridian; *Km* = mean central keratometry; *Kmax* = maximum keratometry; *ISV* = index of surface variance; *IVA* = index of vertical asymmetry; *KI* = keratoconus index; *CKI* = central keratoconus index; *IHA* = index of height asymmetry; *IHD* = index of height decentration; *BAD-D* = Belin/Ambrosio enhanced ectasia total deviation index

^*a*^ Independent samples t-test

### Corneal biomechanics

Comparisons of the main corneal biomechanical parameters between the NAC and AC groups are presented in Table 3. The TBI in the AC group was significantly higher than that in the NAC group (*P* = 0.04). No significant difference was observed in the other parameters between the two groups (*P* > 0.05 for all).

**Table 3:** Comparison of corneal biomechanical parameters between the non-allergic conjunctivitis (NAC) and allergic conjunctivitis (AC) groups

Parameter	NAC	AC	*p*
	Mean ± SD	Mean ± SD	
IOP (mmHg)	16.03 ± 2.40	15.90 ± 1.96	0.84^*a*^
bIOP (mmHg)	15.70 ± 1.99	15.41 ± 1.86	0.61^*a*^
CCT (μm)	555.15 ± 26.73	563.20 ± 27.99	0.32^*a*^
A1 time (ms)	7.60 ± 0.27	7.60 ± 0.23	0.97^*a*^
A1 velocity (m/s)	0.15 ± 0.02	0.15 ± 0.02	0.72^*a*^
A1 length (mm)	2.29 ± 0.31	2.28 ± 0.35	0.94^*a*^
A2 time (ms)	22.44 ± 0.43	22.34 ± 0.34	0.39^*a*^
A2 velocity (m/s)	−0.24 ± 0.05	−0.25 ± 0.03	0.34^*a*^
A2 length (mm)	2.10 ± 0.41	2.05 ± 0.37	0.63^*a*^
DA (mm)	1.04 ± 0.10	1.04 ± 0.08	0.95^*a*^
HC time (ms)	17.43 ± 0.52	17.46 ± 0.35	0.78^*a*^
PD (mm)	4.87 ± 0.33	4.84 ± 0.27	0.74^*a*^
Radius (mm)	7.38 ± 0.81	7.23 ± 0.75	0.52^*a*^
Max DA ratio (2.00 mm)	4.18 ± 0.46	4.15 ± 0.43	0.83^*a*^
Max DA ratio (1.00 mm)	1.55 ± 0.05	1.56 ± 0.05	0.69^*a*^
ARTh	493.36 ± 56.48	486.94 ± 79.36	0.76^*a*^
SP-A1	107.93 ± 15.92	107.93 ± 12.19	0.99^*a*^
	Median (P25, P75)	Median (P25, P75)	
CBI	0.11 (0.00–0.20)	0.02 (0.00–0.07)	0.15^*b*^
TBI	0.04 (0.00–0.14)	0.43 (0.12–0.60)	0.04^*b*^

*NAC* = non-allergic conjunctivitis; *AC* = allergic conjunctivitis; *KC* = keratoconus; *IOP* = intraocular pressure; *bIOP* = biomechanical-corrected intraocular pressure; *CCT* = central corneal thickness; *A1* = first applanation; *A2* = second applanation; *DA* = deformation amplitude; *HC* = highest concavity; *PD* = peak distance; *ARTh* = Ambrosio relational thickness horizontal; *SP-A1* = stiffness parameter at first applanation; *CBI* = Corvis biomechanical index; *TBI* = tomography and biomechanical index

^*a*^ Independent samples t-test

^*b*^ Mann-Whitney U test

### Corneal thickness mapping

Comparisons of the corneal total thickness and epithelial thickness parameters between the NAC and AC groups are shown in Table 4. Although the corneal total thickness parameters were not different between the two groups, the average epithelial thickness in the 2–5 mm and 5–7 mm annuli and the minimum epithelial thickness within 7 mm were significantly thinner in the AC group than in the NAC group (*P* <  0.05 for all). The SD of the epithelial thickness within 7 mm was higher in the AC group than in the NAC group (*P* = 0.01).

**Table 4:** Comparison of corneal thickness and epithelial thickness parameters between the non-allergic conjunctivitis (NAC) and allergic conjunctivitis (AC) groups

Parameter	NAC (μm)	AC (μm)	*p*^*a*^
	Mean ± SD	Mean ± SD	
Corneal total thickness			
2 mm	536.95 ± 24.00	536.20 ± 25.62	0.92
2–5 mm	562.36 ± 23.53	560.78 ± 26.48	0.83
5–7 mm	600.33 ± 26.50	599.13 ± 27.23	0.88
7–9 mm	643.36 ± 31.24	641.08 ± 29.18	0.79
5 mm min	531.05 ± 24.28	530.40 ± 25.36	0.93
5 mm min-max	−64.10 ± 7.22	−63.67 ± 11.87	0.89
Corneal epithelial thickness			
2 mm	52.25 ± 2.24	51.07 ± 3.54	0.19
2–5 mm	52.67 ± 1.91	50.44 ± 3.10	0.01
5–7 mm	52.18 ± 2.32	50.20 ± 3.41	0.03
7–9 mm	50.48 ± 2.66	49.87 ± 3.38	0.50
7 mm min	47.80 ± 3.68	44.47 ± 4.42	0.01
7 mm max	55.60 ± 2.89	55.23 ± 3.84	0.72
7 mm min-max	−8.25 ± 4.91	−10.87 ± 5.21	0.08
7 mm SD	1.53 ± 0.69	2.26 ± 1.10	0.01

*NAC* = non-allergic conjunctivitis; *AC* = allergic conjunctivitis; *SD* = standard deviation; *min* = minimum; *max* = maximum; *min-max* = the difference between minimum and maximum

^*a*^ Independent samples t-test

### Corneal densitometry

Table 5 shows a comparison of the corneal densitometry parameters of different annuli and layers between the NAC and AC patients. The average densitometry values of the total cornea and the anterior layer were higher in the AC group than in the NAC group (both *P* <  0.001).

**Table 5:** Comparison of corneal densitometry parameters between the non-allergic conjunctivitis (NAC) and allergic conjunctivitis (AC) groups

Densitometry	NAC	AC	*p*^*a*^
	Mean ± SD	Mean ± SD	
0–2 mm			
Anterior layer	22.07 ± 1.22	23.12 ± 2.28	0.07
Central layer	13.45 ± 0.95	13.73 ± 0.76	0.26
Posterior layer	9.58 ± 1.01	9.92 ± 0.68	0.16
Average	15.02 ± 0.85	15.57 ± 1.04	0.05
2–6 mm			
Anterior layer	19.60 ± 0.96	20.41 ± 1.66	0.05
Central layer	11.97 ± 0.53	12.27 ± 0.58	0.07
Posterior layer	8.82 ± 0.79	9.13 ± 0.56	0.11
Average	13.55 ± 0.62	13.92 ± 0.76	0.07
6–10 mm			
Anterior layer	18.30 ± 1.65	22.91 ± 4.02	< 0.001
Central layer	11.73 ± 1.01	12.93 ± 1.83	0.01
Posterior layer	9.39 ± 0.79	10.09 ± 1.47	0.06
Average	13.14 ± 1.06	15.31 ± 2.06	< 0.001
10–12 mm			
Anterior layer	27.61 ± 7.33	34.37 ± 6.05	0.001
Central layer	19.24 ± 4.86	18.25 ± 3.27	0.39
Posterior layer	14.13 ± 2.65	13.23 ± 3.67	0.35
Average	20.32 ± 4.55	22.50 ± 3.37	0.06
Total			
Anterior layer	20.79 ± 1.15	23.71 ± 2.40	< 0.001
Central layer	13.26 ± 0.80	13.69 ± 1.18	0.16
Posterior layer	9.96 ± 0.71	10.20 ± 1.10	0.40
Average	14.66 ± 0.72	15.83 ± 1.16	< 0.001

*NAC* = non-allergic conjunctivitis; *AC* = allergic conjunctivitis

^*a*^ Independent samples t-test

### Correlation of altered corneal tomographic parameters with altered corneal biomechanical and epithelial thickness parameters in the AC group

Table 6 shows the correlations of altered corneal tomographic parameters with the TBI and the altered corneal epithelial thickness parameters in the AC group. The TBI was positively correlated with the ISV (*r* = 0.653, *P* < 0.001), IVA (*r* = 0.673, *P* < 0.001), KI (*r* = 0.716, *P* < 0.001), IHD (*r* = 0.612, *P* < 0.001) and BAD-D (*r* = 0.693, *P* < 0.001). The ISV, IVA and KI were negatively correlated with average epithelial thickness in the 2–5 mm and 5–7 mm zones and with the minimum thickness (*P* < 0.05 for all). The ISV and KI were positively correlated with epithelial thickness SD (both *P* < 0.05).

**Table 6:** Correlation of corneal tomographic parameters with biomechanical and epithelial thickness parameters in the allergic conjunctivitis (AC) group

		TBI	Epithelial thickness			
			2–5 mm	5–7 mm	min	SD
ISV	r	0.653	−0.347	− 0.476	− 0.523	0.533
	*p*^*a*^	< 0.001^*^	0.060	0.008^*^	0.003^*^	0.002^*^
IVA	r	0.673	−0.414	−0.439	− 0.450	0.293
	*p*^*a*^	< 0.001^*^	0.023^*^	0.015^*^	0.012^*^	0.116
KI	r	0.716	−0.295	−0.368	− 0.355	0.385
	*p*^*a*^	< 0.001^*^	0.113	0.045^*^	0.054	0.036^*^
IHD	r	0.612	−0.220	−0.255	− 0.353	0.257
	*p*^*a*^	< 0.001^*^	0.244	0.173	0.056	0.170
BAD-D	r	0.693	−0.298	−0.291	− 0.253	0.264
	*p*^*a*^	< 0.001^*^	0.110	0.119	0.178	0.158

*ISV* = index of surface variance; *IVA* = index of vertical asymmetry; *KI* = keratoconus index; *IHD* = index of height decentration; *BAD-D* = Belin/Ambrosio enhanced ectasia total deviation index; *min* = minimum; *SD* = standard deviation; *TBI* = tomography and biomechanical index

^*a*^ Statistically significant findings (*P* < 0.05) are indicated by an asterisk

### Correlation of eye rubbing frequency and ocular allergic sign scores with altered corneal tomographic, corneal biomechanical and epithelial thickness parameters in the AC group

Results from the Spearman correlation analysis of the eye rubbing frequency and ocular allergic sign scores with the corneal tomographic parameters, TBI and epithelial thickness parameters are presented in Table 7. The eye rubbing frequency was positively related to the IVA and BAD-D but negatively related to the average epithelial thickness in the 2–5 mm annulus and the 5–7 mm annulus and with the minimum epithelial thickness within 7 mm (*P* < 0.05 for all). The severity of ocular allergic signs was positively related to the ISV, BAD-D, TBI and epithelial thickness SD but negatively related to the average epithelial thickness in the 2–5 mm annulus and 5–7 mm annulus (*P* < 0.05 for all).

**Table 7:** Correlation of eye rubbing frequency and ocular allergic sign scores with corneal tomographic, biomechanical and epithelial thickness parameters in the allergic conjunctivitis (AC) group

		Corneal tomography					TBI	Epithelial thickness			
		ISV	IVA	KI	IHD	BAD-D		2–5 mm	5–7 mm	min	SD
Eye rubbing	r	0.348	0.362	0.228	0.276	0.424	0.341	−0.648	−0.553	−0.443	0.122
	*p*^*a*^	0.059	0.049^*^	0.225	0.140	0.020^*^	0.065	< 0.001^*^	0.002^*^	0.014^*^	0.522
Hyperemia	r	0.293	0.034	−0.103	0.066	0.084	0.143	0.075	−0.147	−0.337	0.391
	*p*^*a*^	0.115	0.857	0.590	0.729	0.658	0.451	0.695	0.439	0.068	0.033^*^
Swelling	r	0.638	0.295	0.179	0.292	0.291	0.315	−0.122	−0.366	−0.560	0.641
	*p*^*a*^	< 0.001^*^	0.113	0.345	0.118	0.119	0.090	0.521	0.047^*^	0.001^*^	< 0.001^*^
Papillae	r	0.520	0.313	0.330	0.295	0.066	0.105	0.043	−0.189	−0.401	0.467
	*p*^*a*^	0.003^*^	0.092	0.075	0.113	0.729	0.580	0.821	0.317	0.028^*^	0.009^*^
Epithelial disorder	r	0.584	0.340	0.308	0.314	0.401	0.370	−0.318	−0.497	−0.608	0.471
	*p*^*a*^	0.001^*^	0.066	0.098	0.091	0.028^*^	0.044^*^	0.087	0.005^*^	< 0.001^*^	0.009^*^

*AC* = allergic conjunctivitis; *ISV* = index of surface variance; *IVA* = index of vertical asymmetry; *KI* = keratoconus index; *IHD* = index of height decentration; *BAD-D* = Belin/Ambrosio enhanced ectasia total deviation index; *TBI* = tomography and biomechanical index; *min* = minimum; *SD* = standard deviation

^*a*^ Statistically significant findings (*P* < 0.05) are indicated by an asterisk

## Discussion

The etiology of KC remains unclear. Patients with AC were reported to have an increased risk of KC [5]. However, methods for assessing early KC development risk and monitoring the indicators of disease progression in AC patients are still lacking. In this study, we found that the corneal morphology, biomechanics and epithelial thickness were altered in AC patients, as indicated by elevated corneal irregularity and asymmetry, an increased TBI and a thinner corneal epithelium, which were further found to be correlated with the eye rubbing frequency and ocular allergic sign scores. Our results are highly consistent with the findings of Mazzotta [8] who showed that allergic patients with eye rubbing and elevated MMP9 concentrations in tears experience faster KC progression, indicated by greater corrected distance visual acuity decrease, higher Kmax values and a thinner corneal thickness. Furthermore, Kmax worsening was closely related to the severity of the papillary subtarsal response. Both our study and the findings from Mazzotta indicate that allergy and eye rubbing may precipitate the onset and exacerbate the progression of KC.

Progressive corneal thinning and a cone-shaped corneal protrusion are the major characteristics of KC and can be identified by corneal topography examination. However, accurately diagnosing KC in the early stage is still a major clinical challenge. Decreases in corneal biomechanics and thinning of the corneal epithelium have been proposed to occur earlier than tissue loss and topographic alterations in KC patients; thus, measuring the corneal biomechanics and epithelial thickness has been reported to aid in the early diagnosis of KC [15–18, 25–28]. Our findings regarding alterations in the corneal tomography, biomechanics and epithelial thickness in AC patients are consistent with the changes in patients with early KC. The 30 AC patients in our study could not be diagnosed with KC because none had the typical clinical signs specific for KC. However, according to the tomographic criteria for KC diagnosis as described by Asgari [29], including Kmax > 48.0 D, ART-max < 339 μm, I-S value > 1.4 D, BAD-D > 1.6, and posterior elevation, 8 patients in our study met the tomographic criteria for KC. Among them, 1 patient met three criteria (ART-max, I-S value and BAD-D), 2 patients met two criteria, and the other 5 patients met one criterion. According to the TBI cutoff of 0.49 proposed by Sedaghat [30] to distinguish KC eyes from normal eyes, 7 patients met the biomechanical criteria for KC.

By Scheimpflug tomography examination, we found that corneal surface irregularity and asymmetry were significantly increased in AC patients, which was indicated by higher ISV, IVA, KI, IHD and BAD-D values compared to those in the NAC patients. These findings agree with those of previous reports. Gautam and associates [31] found that the corneal surface asymmetry index was significantly higher in VKC children than in normal children. Similarly, Lapid-Gortzak et al. [32] found more abnormal corneal videokeratography patterns in VKC patients, with an increased corneal asymmetry index (inferior-superior asymmetry), an increased corneal irregularity index and increased corneal steepening in VKC patients compared to normal subjects. Although the changes in the corneal tomography of the AC patients in our study were far from the diagnostic threshold of KC, our findings could still serve as supportive evidence for the hypothesis of AC as an etiology of KC.

Our study found that the TBI was significantly greater in the AC group than in the NAC group among all the biomechanical parameters measured by Corvis ST. The TBI is a combined parameter derived from Scheimpflug-based corneal tomography and biomechanical assessments. The TBI has been proven to be useful for the early screening of corneal ectasia and subclinical ectasia with normal topography, and it has shown a high sensitivity and specificity in previous studies [16, 30, 33–35]. Sedaghat and colleagues [30] found that among all the corneal biomechanical parameters measured by the Ocular Response Analyzer and Corvis ST, the TBI had the highest discriminative ability to distinguish KC eyes from normal eyes. The cutoff point was 0.49 for an area under the curve (AUC) of 1.00, and the sensitivity and specificity were both 100%. Similarly, Ambrósio et al. [33] found that the TBI cutoff value was 0.48 for detecting corneal ectasia, with an AUC of 0.996, specificity of 96.2%, and sensitivity of 98.8%. The median TBI of the AC group (0.43) in our study was near the previously derived cutoff points (0.49 and 0.48) for distinguishing eyes with corneal ectasia from normal eyes. This result, to a certain extent, demonstrates the possibility of AC progressing to KC, and the TBI could be used as an indicator of KC development risk in AC patients. Thus, AC patients should be advised to routinely undergo corneal tomographic and biomechanical measurements to evaluate the risk of corneal ectasia development. Moreover, because a younger age is associated with faster KC progression, pediatric and pubertal allergy patients in particular need close corneal tomographic and biomechanical monitoring.

In KC patients, corneal epithelial remodeling and thinning in the cone area have been reported to maintain a smooth corneal surface and mask the already ongoing stromal protrusion, resulting in the delayed detection of KC [28]. Measurement of the epithelial thickness has been proven to aid in the early diagnosis of KC [25–27]. To the best of our knowledge, this is the first study to reveal corneal epithelial thinning and an uneven corneal thickness distribution in AC patients, indicated by a thinner average corneal epithelial thickness with greater variation. Furthermore, we found that the decrease in corneal epithelial thickness was closely related to the eye rubbing frequency in AC patients. The friction between the palpebral conjunctiva and corneal epithelium caused by mechanical eye rubbing leads to epithelial trauma, thinning and remodeling. Consistent with our finding, an 18.4% reduction in both the central and midperipheral epithelial thickness was shown to occur in normal corneas immediately after 15 s of mild to moderate eye rubbing [36]. Furthermore, eye rubbing can increase the release of inflammatory mediators in the ocular surface, which may result in corneal epithelial damage. Previous research has demonstrated that 1 min of eye rubbing significantly increased the tear concentrations of MMP-13, IL-6 and TNF-α in normal subjects and these molecules could potentially cause keratocyte apoptosis [13]. Tear eotaxin concentrations were found to be significantly increased in VKC patients in previous reports [37, 38], inducing eosinophilic infiltration and granular protein release, which in turn exerted toxic effects on corneal epithelial cells and contributed to the breakdown of the epithelial barrier, resulting in corneal epithelial damage [39, 40]. Thus, because eye rubbing has been proposed as a risk factor for precipitating the onset of KC and exacerbating its progression, AC patients should be advised to avoid eye rubbing to prevent KC, especially in high-risk populations, such as young children and patients with severe conditions or a long disease duration.

Additionally, previous studies have shown increased corneal densitometry in KC patients that was correlated with the severity of KC [41]. In our study, we found that the average densitometry values of the total cornea and the anterior layer were higher in the AC patients than in NAC patients. Our findings are consistent with those of a previous study in which an increase in corneal densitometry parameters was observed in both the average of the total cornea and the anterior layer in patients with VKC compared to normal subjects [42]. Corneal densitometry, which is related to corneal transparency, can be influenced by changes in the corneal structure. Corneal microtrauma and uneven thinning induced by eye rubbing lead to corneal irregularities and a loss of corneal transparency. Moreover, increased inflammatory mediator release can also influence corneal transparency through the recruitment of inflammatory cells, as well as their toxic effects on the cornea. Corneal confocal microscopy findings in VKC patients have revealed changes in the corneal microstructure and morphology, including an increased cell diameter, increased cellular activation of the superficial epithelium, and an increased number of inflammatory cells in the epithelium and anterior stroma [43].

Our study has some limitations. The small sample size does not allow us to apply our results to the general population. Studies with larger sample sizes are needed in the future to investigate the relationship between the degree of biomechanical changes and the duration of allergy and to compare corneal biomechanical and morphological changes between different types of AC. Moreover, although we found close correlations between altered corneal topographic parameters and altered corneal biomechanical and epithelial thickness parameters in the AC group, these correlations do not prove a cause-and-effect relationship. In addition, the current investigation is a cross-sectional observational study, and a follow-up study to explore corneal biomechanical changes over time and to identify risk factors for progression in AC patients will be necessary. Furthermore, Scheimpflug-based air-puff devices such as Corvis ST have limitations in detecting corneal biomechanics, as corneal biodynamics is also affected by several other parameters including white-to-white distance, mean curvature, pachymetry, scleral connection and IOP. Therefore, devices that can directly and effectively measure the corneal elastic module are required.

## Conclusions

The findings of this study indicate that ophthalmologists and pediatricians should recommend their patients with AC to routinely undergo corneal tomographic and biomechanical measurements for early KC screening. Moreover, eye-rubbing, allergy patients should be closely monitored, especially those near puberty who are at higher risk for ectasia development and progression. Since alterations in the corneal morphology, biomechanics and epithelial thickness are correlated with eye rubbing frequency and the severity of ocular surface signs in AC patients, these patients should be advised to cease eye rubbing to reduce the risk of KC occurrence. Additionally, it is necessary to standardize the treatments for allergies and inflammation. These treatments include topical antihistamine agents, mast cell stabilizers, steroids, preservative-free lubricants, and immunosuppressive agents such as cyclosporine A or tacrolimus in severe cases with giant papillary response or VKC [44, 45]. KC patients with allergy and eye rubbing who are at a higher risk of progression at corneal tomography and biomechanics are recommended to undergo timely preventive measures, specifically corneal collagen cross-linking, without waiting for further progression [46].

## Data Availability

The datasets used in the current study are available from the corresponding author upon reasonable request.

## Abbreviations

AC: Allergic conjunctivitis
NAC: Non-allergic conjunctivitis
KC: Keratoconus
MMP: Matrix metalloproteinase
IL: Interleukin
IFN: Interferon
TNF: Tumor necrosis factor
VKC: Vernal keratoconjunctivitis
CCT: Central corneal thickness
K1: Keratometry of the flattest meridian
K2: Keratometry of the steepest meridian
Km: Mean central keratometry
Kmax: Maximum keratometry
ISV: Index of surface variance
IVA: Index of vertical asymmetry
KI: Keratoconus index
CKI: Central keratoconus index
IHA: Index of height asymmetry
IHD: Index of height decentration
BAD-D: Belin/Ambrosio enhanced ectasia total deviation index
IOP: Intraocular pressure
bIOP: Biomechanical-corrected intraocular pressure
A1: First applanation
A2: Second applanation
DA: Deformation amplitude
HC: Highest concavity
PD: Peak distance
ARTh: Ambrosio relational thickness horizontal
SP-A1: Stiffness parameter at first applanation
CBI: Corvis biomechanical index
TBI: Tomography and biomechanical index
SD: Standard deviation
AUC: Area under the curve
